# Current Management of Membranous Urethral Strictures Due to Radiation

**DOI:** 10.3389/fsurg.2021.635060

**Published:** 2021-03-04

**Authors:** Marjan Waterloos, Francisco Martins, Wesley Verla, Luis Alex Kluth, Nicolaas Lumen

**Affiliations:** ^1^Department of Urology, AZ Maria Middelares Ghent, Ghent, Belgium; ^2^Department of Urology, University Hospital Ghent, Ghent, Belgium; ^3^Department of Urology, University of Lisbon School of Medicine, Lisbon, Portugal; ^4^Department of Urology, University Hospital Frankfurt, Frankfurt am Main, Germany

**Keywords:** urethal stricture, radiotherapy, membranous urethral stricture, radiation-induced, urethroplasty

## Abstract

Radiotherapy is a frequently used treatment for prostate cancer. It does not only causes the intended damage to cancer cells, but also affects healthy surrounding tissue. As a result radiation-induced urethral strictures occur in 2.2% of prostate cancer patients. Management of urethral strictures is challenging due to the presence of poor vascularized tissue for reconstruction and the proximity of the sphincter, which can impair the functional outcome. This review provides a literature overview of risk factors, diagnostics and management of radiation-induced urethral strictures.

## Introduction

Prostate cancer is the second most frequent cancer in men. Treatment options in localized prostate cancer are active surveillance, surgical treatment or radiation therapy (External Beam Radiotherapy EBRT, Brachytherapy BT or combination of both).

Radiation therapy for prostate cancer is the chosen treatment in approximately 25–34% (1, 2).

Radiation causes ionization events and production of free radicals resulting in different types of DNA damage, eventually leading to vascular injury (endarteritis) and stem cell damage. This leads to atrophy and poorly oxygenated tissue with eventual tissue scarring (3). While intended in cancer cells, it also affects healthy tissue, resulting in a range of side-effects and pathology.

Radiation induced urethral strictures usually occur at the bulbomembranous urethra, even though theoretically receiving lower radiation dose (4).

Hughes et al. examined the specimens of patients who underwent a urethroplasty for a membranous stricture and found that post-radiation specimens had a significantly decreased vascularity compared to specimens of non-radiated etiology (5).

The management of radiation induced strictures remains challenging. It differs from non-radiotherapy related strictures by the scarred tissue with reduced healing capacity. Due to the proximity of the sphincter functional outcome may be impaired (6).

Since the high rates of curation or disease control of prostate cancer nowadays, quality of life is very important to consider in the treatment of these strictures (1).

For the purpose of this review we searched the pubmed library from the year 2000 to 2020.

## Epidemiology, Etiology, and Risk Factors

The prevalence of radiation induced urethral strictures in prostate cancer patients is 2.2% at a median follow-up of 4 years: 1.5% in patients undergoing External Beam Radiotherapy (EBRT), 1.9% in patients undergoing Brachytherapy (BT) and 4.9% in patients receiving a combination of both EBRT-BT. When EBRT is used as a salvage treatment stricture incidence increases to 3–10% (1, 6, 7).

Stricture incidence will increase with time, in contradiction with strictures after radical prostatectomy (8, 9). Median time to stricture formation is estimated between 2.2 and 3.4 years after radiation therapy (1). The CaPSURE database revealed a stricture rate of 1% directly after treatment to 16% after 4 years (7).

A systematic review of Awad showed no difference in urethral stricture development concerning age, proportion of patients on Androgen Deprivation Therapy (ADT) and biochemically equivalent dose (BED) (1). This last observation is also found in the ASCENDE-RT trial, where only little correlation between urethral stricture and dose to the prostate was found (10). Other studies (case series, case control series) demonstrated a clear dose-related effect on urinary morbidity (11–13). Hindson et al. reports an increased stricture rate when radiotherapy was separated in 2 sessions, in comparison of 3 and 4 treatments (14).

In brachytherapy the region inferior to the apex is commonly referred to as “the hotspot” (15). Decreasing the radiation dose to the hot spot, special care during BT-needle placement, avoiding midline insertions, and using plastic needles instead of steel needles, have shown to be effective measures to reduce the rate of urethral strictures (1).

Multiple studies demonstrated clearly an increased risk of urethral stricture in patients who had a TURP prior to radiation therapy. Underlying mechanism is thought to be devascularization of the urethra after TURP in combination with mucosal impairment due to radiation damage (4, 16, 17).

It remains controversial whether combination with hormonal therapy increases the risk of urinary morbidity (11, 18). According to the CaPSURE database there was no change in stricture rate therapy when ADT was associated to another treatment (7). This was also the conclusion in the systematic review of Awad (1).

## Diagnostics

Diagnostic workup is important for planning of the surgical intervention, and can be tailored on a case per case base.

Patients with radiation-induced strictures will present more often with storage lower urinary tract symptoms (LUTS) as a side-effect of their prior radiotherapy treatment. It can be important to determine the pre-operative bladder function by performing a urodynamic study. In other cases uroflowmetry will be sufficient. Radiographic evaluation of the length and location of the stricture is necessary. When a retrograde urethrogram (RUG) is insufficient to evaluate the bladder neck a voiding urethrocystogram (VCUG) can be performed (6).

According to the SIU/ICUD consultation on urethral strictures diagnostic workup for posterior urethral stricture should consist of history, physical examination, laboratory investigations (urine, renal function, prostate-specific antigen), uroflowmetry an postvoid residual volume, cystoscopy and antegrade cystoscopy when evaluation of the anatomy proximal of the stenosis is needed. On indication a retrograde urethrography, voiding cystourethrography, prostate and upper urinary tract imaging or urodynamic evaluation can be performed (2).

## Treatment

### Conservative

In case surgical management is not useful or feasible, chronic urinary catheter will allow urinary drainage. A chronic suprapubic catheter can be a viable option in frail or therapy refractory patients with complete urethral obliteration (19).

Incontinence can be a predominant feature even in patients with urethral strictures. Conservative options for incontinence include penile clamp, condom catheter, and use of sanitary pads (20).

In a study of Fuchs urinary diversion is also used as a measure to obtain urethral rest prior to reconstructive surgery. At a follow-up period of 6 months 49% of the patients preferred to keep their chronic suprapubic tube, instead of undergoing a urethroplasty (21).

All complications related to chronic urinary drainage, such as irritative symptoms, bladder pain, infection and stone formation should be taken in consideration.

### Endoluminal

Even in non-radiation related strictures endoluminal treatment has poor results, especially in longer and high grade strictures. Due to impaired tissue quality the outcome in radiation-induced strictures is even poorer. When there is a complete obliteration of the urethral lumen, endoluminal treatment is contra-indicated.

Brandes et al. reports different results after Direct Vision Internal Urethrotomy (DVIU) or dilatation according to the treatment modality: stricture recurrence time of 3.7, 26, and 10.9 months after BT, EBRT and combination BT-EBRT, respectively. Total success rate at 4 years follow-up is 20% with EBRT and 0% with BT, concluding to endoluminal treatment as a palliative option (22). Chen et al. demonstrated a stricture recurrence rate of 50% within 16–60 months after DVIU or dilatation (23).

Sullivan et al. studied a relatively large cohort of patients treated with brachytherapy, followed by endoluminal treatment and a recurrence rate of 49% was reported at a median follow-up of 16 months (4).

Merrick reports a higher patency rate of 69% in a retrospective case series (13).

To stabilize fibrosis after endoluminal treatment intermittent self-dilatation (ISD) can be attempted (6). This should be considered as a palliative treatment, in patients who are unwilling or unable to undergo more invasive surgical strategies (4, 13, 24). On the other hand some authors state that repetitive endoluminal treatment might induce further fibrosis (25).

The conclusion of a study of Lubahn about quality of life in patients performing ISD, states that it is inappropriate to implement ISD in patients that are still amenable for reconstruction, since it's associated with a decrease in quality of life (26).

### Open Reconstruction

#### Excision and Primary Anastomosis

This technique will provide a durable long-term outcome, when surrounding scarred tissue is resected and a tension-free anastomosis can be achieved (Table 1).

**Table 1:** Urethroplasty for radiation-induced strictures.

	**Urethroplasty technique**	***N***	**FU (years)**	**EBRT**	**BT**	**EBRT/BT**	**Other**	**Time to stricture development (years)**	**Mean stricture length (cm)**	**Patency rate (%)**	**Time to recurrence (months)**	**New onset incontinence (%)**	**Deterioration erectile function (%)**
Rourke et al. (27)	EPA	23	4.25	20	15	NR	0	4.9	2.1	91	29.8	26	35
	Graft/Flap	12							6.1	75		25	0
Hofer et al. (28)	EPA	66	3.5	28	28	9	1	6.4	2.4	70	10.15	36	7
	Graft/Flap	6	5.5	5	1	0	0	13.05	4.3	83	7	50	NR
Glass et al. (29)	EPA	22	3.3	11	4	7	7	7	2.6	95	12	NR	NR
	BMG	5								80			
	Flap	2								50			
Meeks et al. (30)	EPA	24	1.75	15	7	6	NR	9.3	2.9	73	5.1	50	3
	BMG	2											
	Flap	4											
Fuchs et al. (31)	EPA	72	2.8	33	26	9	4	6	2.3	76	4.2	35	NR
Policastro et al. (32)	BMG	79	1.75	36	13	10	20	4	3	82.3	5	8.1	NR
Vetterlein et al. (33)	BMG	47	3.6	33	5	8	1	NR	NR	67	3	NR	NR

*EPA, Excision and Primary Anastomosis; BMG, Buccal Mucosa Graft; FU, Follow-Up; EBRT, External Beam Radiotherapy; BT, Brachytherapy; NR, Not Reported*.

Rourke published a case series of 35 patients, in which EPA was performed in 65.7% of the cases, and in the other cases buccal mucosa or penile skin flap was used for substitution urethroplasty. All patients had failed prior endoscopic treatment. Strictures treated with EPA and substitution urethroplasty had a mean length of 2.1 and 6.1 cm, respectively. They were all located at the bulbomembranous urethra. Patency rate after follow-up of 4 years was 91% for EPA and 75% for substitution urethroplasty.

One out of four patients complained of worsening or new onset of urinary incontinence, of which 50% had a prior TURP.

In total 68.6% of patients reported a change in continence, erectile function or voiding function after treatment, even when an unobstructed urethra was achieved. This last finding is most likely related to radiotherapy-induced bladder dysfunction (27).

Hofer et al. demonstrates a success rate of 70% with EPA in a group of 66 patients, with mean stricture length of 2.4 cm. *De novo* postoperative urinary incontinence was reported in 36% of the cases. Strictures longer than 2 cm were associated with a greater risk of incontinence.

New onset erectile dysfunction was reported in only 7% of the patients. Stricture location or length was not associated with erectile function (28).

A subsequent cohort from the same group a few years later showed an improved success rate of 85%, attributed to increased surgeon experience. There was a decreased incontinence rate, however presentation of more severe urinary incontinence. Risk of recurrence was not associated with the length of follow-up, concluding that recurrence occurred in the early postoperative period (31).

In a study of Glass et al. 29 patients were treated with EPA (76%), buccal graft urethroplasty (17%) and perineal flap urethroplasty (7%) for radiation-induced strictures with a median length of 2.6 cm. An overall success rate of 90% was reported. Outcome on continence and erectile function was not reported (29).

In another case series of Meeks et al. 30 patients underwent urethroplasty for radiation-induced strictures, all had previous failed endoscopic treatments. Overall patency rate after EPA (84%) and substitution urethroplasty (16%) was 73%. Follow-up was only 21 months. Urinary incontinence after surgery occurred in 50% of the patients. There was no significant change in erectile function (30).

Elliott et al. reports a success rate of 72% after urethroplasty for strictures after prostate cancer treatment, however this was a very heterogenous cohort and there was a wide range of stricture etiology and surgical techniques. Again, radiation therapy was suggested as an important predictive factor for stricture recurrence. An algorithm was developed, in which long radiation (EBRT) induced strictures are advised to be treated with perineal urethrostomy instead of other reconstructive techniques (flaps or two staged procedures) (34).

Higher urinary stress incontinence rates are reported when EPA is performed for radiation-induced strictures (33%), in comparison to pelvic fracture related injuries (12%) in a small retrospective case series of Chung (35).

#### Substitution Urethroplasty

Even more than in the EPA technique, urethroplasty using grafts or flaps is impaired by the poor quality of the irradiated surrounding tissue. Substitution urethroplasty is used for longer strictures and when EPA is no longer feasible (Table 1).

In a retrospective cohort of Vetterlein et al. 47 patients underwent buccal mucosa ventral urethroplasty. Mean graft length was 5 cm. A recurrence rate of 33% was observed. In this study validated questionaires (USS-PROM) were used to evaluate patient reported outcomes. Postoperatively 53% patients reported daily urinary incontinence, and 26% required an artificial urinary sphincter implantation. Erectile dysfunction or absence of sexual activity was present in almost all of the patients (33).

In the case series of Hofer et al. 6 patients with a median strictyure length of 4.25 cm were treated with substitution urethroplasty. Only one patient had a recurrence at 5.5 years follow-up. New onset urinary incontinence was present in 50% of the patients. There was no change in erectile function after surgery (28).

Palmer describes ventral onlay buccal mucosa urethroplasty and use of a gracilis muscle flap for long segment complex strictures. The gracilis muscle flap provides a well-vascularized graft bed for the buccal graft. Mean stricture length was 8.2 cm and in 9 of the 20 patients stricture etiology was radiotherapy. A patency rate of 80% was achieved at a mean follow-up of 40 months. Mean time to stricture recurrence was 10 months (36).

A multi-institutional retrospective series of dorsal onlay buccal mucosa urethroplasty in 79 patients, showed a patency rate of 82.3%, and a *de novo* incontinence rate of 8%. There was a short median follow-up of 21 months (32).

### Urinary Diversion

When there are no more reconstructive options left and patients have a refractory bladder outlet obstruction or other severe symptoms such as uncontrollable pelvic pain, urinary diversion can be discussed.

In a case series of Sack et al. 15 patients with previous radiotherapy and/or cryotherapy were treated with surgical extirpation and urinary diversion for different radio- or cryotherapy induced problems including urethral strictures. There were on average 3.7 failed previous interventions. Surgical extirpation (cystectomy or cystoprostatectomy) was performed and urinary diversion was accomplished by ileal conduit, catheterizable pouch or colon conduit. Perioperative morbidity was higher than in a non-irradiated population. Postoperative quality of life (QoL) was measured, and patients reported a satisfying outcome and would have undergone the surgery sooner (37).

Al Hussein Al Awamlh et al. also reports a significant improve in QoL, despite perioperative complication risks, in patients with severe radiotherapy related complications (fistulas, radiation cystitis, pelvic pain or incontinence) (38).

In case of preserved bladder capacity bladder preservation can be attempted, with closure of the bladder neck and continent urinary diversion (20).

## Discussion

Radiotherapy induces oxidative stress, resulting in an effective cancer treatment as a short term result. However, on the long term it causes chronic inflammation and micro-angiopathy, resulting in tissue damage. This late side-effect explains the potential late onset of radiation-induced complications.

No studies so far were able to demonstrate a firm correlation between urethral strictures and urethral dose of radiation. However, more profound dosimetric studies should be performed to support this conclusion.

The management of radiation-induced urethral strictures is complicated due to several reasons: the proximity of the external urethral sphincter since most of these strictures are located in the bulbomembranous urethra, the poor quality of local tissue needed for reconstruction and impaired vascularity that will lead to a difficult wound healing process (25, 39).

Literature is still limited and most studies are small retrospective case series. As a result of this consideration as a late onset complication, a significant amount of studies has a high rate of loss to follow-up, possibly underestimating the prevalence.

Conservative management can be an option in frail patients, or when reconstructive surgery is no longer a viable option, and usually consists of chronic urinary drainage. Chronic catheter related problems should be taken into account.

Endoluminal treatment has a success rate between 0 and 51%, based on retrospective case series (4, 13, 22, 23). A single endoluminal treatment can be attempted since it has an acceptable patency rate and a much lower incontinence rate than open reconstruction. On the contrary repetitive DVIU or dilatation might provoke further fibrosis of the radiated tissue and can lead to a delay of more reliable reconstructive options. Intermittent selfcatheterization can be used as a palliative treatment, when no other reconstructive options are left (4, 6, 13, 24). However, it is often associated with a lower quality of life (26).

Excision and primary anastomosis of radiation-induced strictures provides durable long term results, with patency rates up to 90%. Most authors also emphasize the feasibility of this technique in most of the cases, provided the stricture is short enough to allow tension-free anastomosis.

For longer strictures, substitution urethroplasty must be performed. Although even more prone to the radiation induced reduction of tissue quality then EPA, long term success rates up to 84% are reported, in small case series. Since this technique is used less frequently then EPA, all studies consist of small case series, so results must be interpretated with caution.

When primary reconstructive techniques fail or concomitant severe symptoms are present, urinary diversion with or without extirpation should be discussed with the patient. Depending on the residual bladder function continent or incontinent diversions can be considered (20). These procedures have a higher complication rate in patients who underwent radiotherapy (37).

After endoluminal treatment a new onset urinary incontinence rate of 10% was reported (4, 13, 22, 23).

Deterioration or new onset of urinary incontinence after urethroplasty for radiation-induced strictures (EPA and substitution urethroplasty) is present in 11–50% of the patients. Incontinence can be mild but a minority of patients will need an artificial urinary sphincter. Incontinence rates are higher after urethroplasty for radiation-induced strictures in comparison to other etiology (35).

Most of the studies report mainly unaltered erectile function after the treatment of radiation-induced strictures (28, 30). This is probably due to the high rates of erectile dysfunction present prior to surgery as a result of the radiotherapy itself. The cavernous nerves located dorsally to the posterior urethra are preserved during some techniques of substitution urethroplasty in contrast to EPA, however this doesn't seem to influence the already low deterioration in erectile function postoperatively.

Concerning the complications a limitation in almost all of these studies was a lack of validated questionnaires to evaluate patient reported outcome measures.

Even when a radiation-induced stricture is successfully treated patients can experience persistent symptoms due to radiation toxicity, for example impaired bladder capacity due to radiocystitis.

## Conclusion

Management of radiation induced urethral strictures remains challenging, with an uncertain outcome and a significant amount of side-effects. Experienced operative skills with good knowledge of all the techniques are required to increase the chance of a good long-term outcome. Quality of life is important to take into account, especially since the prognosis of prostate cancer has been improved over the last decades.

Patients should be informed that returning to a urological situation prior to their prostate cancer treatment is not a realistic expectation, since radiation-induced bladder dysfunction can impair outcome of reconstructive surgery.

## Author Contributions

All authors listed have made a substantial, direct and intellectual contribution to the work, and approved it for publication.

## Conflict of Interest

The authors declare that the research was conducted in the absence of any commercial or financial relationships that could be construed as a potential conflict of interest.
